# Items

**Published:** 1882-07

**Authors:** 


### Items.

---

CUFFEE AND THE PHILADELPHIA DOCTORS—THE BORRIO-BOOLA-GHA MISSION REVIVED.—At a meeting of medical teachers and practitioners, called to confer with J. Berrien Lindsley, M.D., LL.D., of Tennessee, in regard to the interests of a medical college for colored students, at Nashville, and held at the Hall of the College of Physicians of Philadelphia, May 8th, 1882, with Prof. S. D. Gross in the chair, Dr. Lindsley stated (*Reporter*) that his object was to gain the endorsement of representative physicians in the North, for the plan of creating a National Board of Trust, chartered by Congress, for the medical education of colored people, which should be empowered to promote this most desirable object, by aiding the most promising educational establishment of the kind in the South, and further defraying the necessary expenses of those young persons of African descent who should display superior intellectual capacity in northern or European schools, as might best serve the great end in view, the elevation of the negro race. It being further designed that special attention should be paid to the complete preparation of of colored physicians as missionaries and explorers in Africa.

Dr. H. Hartshorne highly approved of the proposed plan as explained by Dr. Lindsley.

Dr. Edward Hartshorne considered Nashville a great intellectual missionary centre, and that if we could establish a good medical school there, the best colored men from the whole southwest would flock to it, and from these the selected few who developed in advance of their race could be sent, on this well devised plan, to northern colleges, where the best possible medical training could be secured for them. *Sic.*

Prof. James Tyson observed that his study of the important subject of medical education had rendered him opposed to the erection of any new colleges, but he did not entertain the same objection to fostering an already established institution. For the last four years colored students have matriculated at the University of Pennsylvania, and one of these graduated the past year with an average as high as that of three-fourths of the class. Another, who will probably graduate next year, has already passed in chemistry, with the unusual average of 98 in a possible 100. Only one white student is ever reported to have left on account of the admission of these colored men, so that the question is practically settled in regard to the University of Pennsylvania, as also it is in the affirmative in Harvard University.

Dr. J. Cheston Morris believes that, having lived, formerly, on a plantation in one of the Southern States, he was intimately acquainted with the black man's capacities, and he was satisfied that for the next 20, 40, or perhaps 100 years, but a small proportion of the negroes could acquire the preliminary training necessary for a great physician.

The Chairman, Prof. Gross, remarked, that "if the negro race could develop a great physician in a hundred years, it would prove its vast superiority to the whites, who had required some two thousand years to accomplish the same feat. He believed that the blacks had capacities which in time will develop to a very high degree, but for the present the negro must have his own schools. Applicants had always been refused at the Jefferson Medical College, and it would probably be a long time before they gained admittance, if they ever did so; he, therefore, cordially approved of the plan of Dr. Lindsley."

And, now, let congress feather the nest of the philanthropist, let "those young persons of African descent" have their expenses paid at "Northern or European colleges" by the government and then let those same young persons clip their ears in grateful recognition of the favors.

---

Dr. Oliver Wendell Holmes, in speaking of the discovery of anæsthesia (*Boston Med. and Surg. Journal*), says:

" Its cradle was the Massachusetts General Hospital as surely as Faneuil Hall was the cradle of liberty. Not that the earliest hint of either of them was first breathed under those roofs. The Messiah was long promised and expected, but he was born and cradled at Bethlehem.

" In spite of the not infrequent attempts to appropriate elsewhere the credit of this heaven-sent gift to mankind, of which the most extraordinary is that of the late Sir James Y. Simpson, in the eighth edition of the *Encyclopædia Brittanica*, quietly superseded, I am pleased to say, in the ninth edition of the same work, Boston is the Bethlehem of this divine birth, and will, in the light of that fact, remain one of the sacred shrines of humanity as long as the waters wash her feet and the winds blow over her dwellings."

For cool, sublime assurance this declaration from "the hub" is typical. But, it should not be forgotten that the speaker is a poet; and poets, by common consent, enjoy a large liberty of expression for the sake of euphony merely. They often take a broad license to make their verses rhyme, not always with fact, but with one another.

---

At a meeting of the Homœopathic Medical Society of Lancaster county, Penn., the following resolution was unanimously adopted:

"*Resolved*, That it is the sense of this meeting that since the practice of homœopathy has established for itself an honorable position in the estimation of the community against all the opposing forces that the Old School could bring to bear against it, there is no advantage or prestige to be derived by homœopathic physicians in consulting with *allopaths*, and, therefore, the recent action of the *Allopathic* Medical Society of the State of New York, in resolving in future to consult with them, was entirely gratuitous."

By way of comment on this act, the *Medical Press and Circular* says:

"Truly, the profession in New York seem to be insensible to any consideration save that 'hankering after the flesh-pots' which has its origin in consultation fees. We are well satisfied that the homœopaths have flung back in the teeth of the New York Medical Society its mean, self-interested patronage, which, indeed, would do no honor even to such people as eclectics and other quacks."

---

THE NEW YORK TRIBUNE of June 9th, contains interviews with Drs. Nathan Bozeman, Frank H. Hamilton and Alonzo Clark respecting the action of the American Medical Association in rejecting the delegates from the New York State Society. These gentlemen approved the view taken of the question by the Association and they all confidently expect the State Society to rescind its hasty legislation at the next meeting. Dr. Bozeman very properly claims that injustice has been done the New York specialists by many medical journals in the wholesale charges brought against them to the effect that they were actuated by mercenary motives and are responsible for the changes in the code. The majority of specialists, he says, are innocent of blame in the matter, but he does not, by any means, undertake to exculpate the few who participated in the *coup d'etat*.

---

OUR friend Dr. S. H. Stout, formerly a resident of this city, and during the late civil war the medical director of the army of Tennessee, has removed to Texas and has assumed the editorial management of the *Comanche Chief*, a weekly newspaper, published in the town of Comanche.

Dr. Stout will practice his profession in his new home, in addition to his recently accepted responsibilities, and the savage suggestious which cluster around the title of his paper need not alarm his neighbors, for it is from gentle manners that his chief glory flows.

Since the above was written, we have been favored with another copy of *The Chief.* For the compliment to THE REGISTER, the editor and the publisher, contained therein, we return our most appreciative thanks, and shall strive to merit the friendship so kindly expressed which comes, unexpectedly to us, from the "Lone Star State."

---

A PHYSICIAN of Buffalo, N. Y., was called to see a man who had, or pretended to have, a severe attack of dysentery. To the surprise of his attendant, the man seemed to grow worse. After three or four days, the friends of the sick man reported, early one morning, that he had died during the night, and applied for a certificate setting forth that fact and the cause thereof. Not suspecting any fraud, the doctor, cheerfully—that is with as much cheerfulness as the circumstances warranted—gave the desired certificate. It subsequently came to light that the patient did not die at all, but was spirited away; only a second-hand corpse was interred in his stead, and all for the purpose of defrauding one little insurance company.

---

AN English exchange complains that Mr. Gladstone's achievements in connection with his rearrangement of taxation in the United Kingdom "press heavily upon an already over-burdened class of professional bread-winners." The increase of tax on carriages is the cause of the comment, as it is claimed that the doctor's carriage is an important, almost indispensable part of his business machinery, and the profession of Great Britain are called upon to exert their combined influence with the members of the House of Commons in the hope of obtaining their opposition to the proposal.

Our English brethren seem to be very sensitive these days to taxation—taxation at home!

---

THE *American Journal of Stimulants and Narcotics* is the latest journalistic venture. It is a monthly magazine, published in New York, and "devoted to a scientific study of acute and chronic poisoning by alcoholic and narcotic agents." Dr. H. H. Kane is the editor, and "The Editors DO hold themselves responsible for all the articles that appear in these pages."

---

DR. H. S. GUTHRIE of Higginsport, reports in the *Ohio Medical Journal*, a case of lead poisoning in a young lady, aged nineteen years, by the daily application of carbonate of lead as a cosmetic to the hands and face, and cured by the use of arsenic. Five drops of Fowlers solution, three times a day, was the preparation and the quantity used.

---

THE recognition of Dr. A. W. Calhoun's fitness for the chairmanship of the Section of Ophthalmology, Otology and Laryngology of the American Medical Association was a deserved compliment to a worthy gentleman and an accomplished physician. We heartily congratulate the doctor upon this evidence of merited appreciation.

---

THE REGISTER takes great pleasure in acknowledging the kind remembrance and courteous attentions, in connection with the recent meeting of the American Medical Association, of Drs. Robert Battey and A. W. Calhoun and, also, of Dr. Talbot Jones, of Saint Paul.

---

AN effort will be made during the next session of the legislature to have the name of Dirt Town (Ga.) changed. This is as it should be. It will be regarded as a commendable step toward estheticism. We wish the movement speedy and complete success.

---

THE University, of Pennsylvania, graduates negroes, and apparently exults in her mixed classes. It is a happy medical college, that, in these days of glorious liberty and rapid progress, can boast a variegated alumni. *De gustibus non disputandum est.*

---

THE *Cincinnati Lancet and Clinic* is not quite as particular as it might well be to give due credit for borrowed items. Speaking of this peculiarity, however, we might, with truth, name several journals.

---

DR. C. R. AGNEW, a celebrated specialist of New York city, was the chief apostle and principal advocate of the new code of New York at Saint Paul. Our readers are already informed of the failure of his mission.

---

THE Jefferson Medical College, Philadelphia, refuses to receive negro students, and it is doubtful if she will ever relax this rule. She does not believe in "black and tan" classes.

---

A CONTRIBUTOR to the *Medical and Surgical Reporter*, extols the value of the elastic bandage in the treatment of fracture of the patella.

---

It is said that a mixture of protoxide of hydrogen and spiritus vini gallici is used with gratifying results in polydipsia.

---

DR. CLIFFORD D. PARKE, of Selma, has been elected president of the Medical Association of the State of Alabama.

---

WANTED.—The February No., 1882, of THE REGISTER. Will pay the retail price for it. Address the Publisher.

---

ETHER SPRAY to the back of the neck is recommended for the vomiting of pregnancy.

